# The Influence of Plant Species Composition on an Endangered Grassland Specialist Reptile, the Hungarian Meadow Viper

**DOI:** 10.1002/ece3.73579

**Published:** 2026-04-28

**Authors:** Mátyás Budai, Gergő Rák, Bálint Wenner, Attila Móré, Barnabás Bancsik, Bence Nagy, Gergő Kovács, Márton Szabolcs, Zsolt Ladnyik, Csaba Molnár, Zsófia Eszter Guller, Attila Lengyel, Csaba Vadász, Edvárd Mizsei

**Affiliations:** ^1^ Juhász‐Nagy Pál Doctoral School of Biology and Environmental Sciences, Faculty of Science and Technology University of Debrecen Debrecen Hungary; ^2^ Centre for Metagenomics University of Debrecen Debrecen Hungary; ^3^ Conservation Ecology Research Group, Institute of Aquatic Ecology HUN‐REN Centre for Ecological Research Budapest Hungary; ^4^ Kiskunság National Park Directorate Kecskemét Hungary; ^5^ Department of Systematic Zoology and Ecology, Faculty of Science Eötvös Loránd University Budapest Hungary; ^6^ Department of Nature Conservation and Landscape Management Hungarian University of Agriculture and Life Sciences Gödöllő Hungary; ^7^ Department of Ecology University of Szeged Szeged Hungary; ^8^ Independent Researcher Hungary; ^9^ Doctoral School of Natural Sciences Hungarian University of Agriculture and Life Sciences Gödöllő Hungary; ^10^ Institute of Ecology and Botany, HUN‐REN Centre for Ecological Research Vácrátót Hungary

**Keywords:** animal conservation, density, habitat drainage, occupancy, plant traits, species richness, vegetation structure

## Abstract

The Hungarian meadow viper ( *Vipera ursinii rakosiensis* ) is one of the most threatened vertebrates in Hungary, whose populations are not growing significantly despite enormous conservation efforts. Previous studies suggested an influence of vertical vegetation structure on habitat use, while the role of horizontal vegetation structure is still poorly understood. In the present study, we used vegetation survey data to investigate the effects of variables related to the horizontal structure and functional composition of vegetation on the occupancy and density of the Hungarian meadow viper. During a spring survey period, we collected viper occurrence data in 59 sampling quadrats alongside plant community samples, then used single‐season occupancy models and N‐mixture models for analysis. After model selection, the best models included the moisture‐related vegetation gradient, species richness, graminoid‐forb ratio, and height of plants as explanatory variables for both occupancy and density. Wetter meadows with fewer plant species, a higher graminoid/forb ratio, and habitats with characteristically lower‐growing plant species were more probable to be used by the vipers. Our results suggest that the horizontal structure of the vegetation influences the habitat use of vipers and also draw attention to the threats posed by more frequent droughts and heatwaves.

## Graphical abstract

The study investigated how the horizontal structure and functional composition of grassland vegetation influence the occupancy and density of the highly endangered Hungarian meadow viper. Using plant community data and viper surveys from 59 quadrats, the best models showed that vipers prefer wetter meadows with fewer and shorter plant species, and a higher graminoid‐to‐forb ratio. These findings highlight the importance of vegetation structure in viper habitat use and the potential risks posed by increasing droughts and heatwaves.

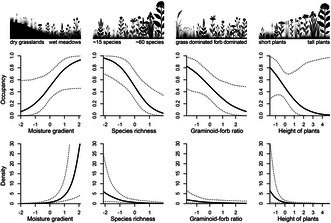

## 1 Introduction

Reptile populations are declining globally (Todd et al. 2010) as approximately 20% of the species are at the brink of extinction (Böhm et al. 2013; Cox et al. 2022). Habitat loss and degradation, introduction and distribution of invasive species, diseases, environmental pollution, overexploitation of habitats and climate change are among the most important factors causing global declines in reptile populations (Gibbons et al. 2000; Farooq et al. 2024). In addition to the innate value of reptiles, their role in ecological systems is not negligible; therefore, their decline may indicate larger‐scale changes (de Miranda 2017). The extinction risk of reptiles is usually higher in tropical areas (Böhm et al. 2013); however, species from temperate zones, such as Europe, may be exposed to the negative effects of the globally warming climate as well (Araújo et al. 2006). Habitat specialists, especially those with a limited range size and habitats that are easily accessible to humans, are particularly vulnerable (Böhm et al. 2016).

When developing conservation strategies, practices must be based on scientific evidence and recommendations from researchers (Sutherland et al. 2004). Policy‐driven conservation can mitigate the positive effects of the implemented methods or can even have negative effects on the ecosystem (Svancara et al. 2005). Information on demographic data and population trends (Conde et al. 2019) and identifying factors influencing the habitat selection of animals has particular importance in ecology and conservation biology (Wecker 1964). In the case of endangered species with low detectability, special modelling methods, such as occupancy modelling and N‐mixture models, can be useful tools for estimating the habitat preferences of these cryptic species (Durso et al. 2011; Ward et al. 2017; Mizsei, Budai, Wener, et al. 2023).

Plant species composition and diversity are considered important features that influence the habitat choices of animals due to their significant effect on ecosystem functioning and the habitat structure they provide (Hooper and Vitousek 1997). Literature on plant species composition and its effect on reptile populations and communities remains scarce; however, earlier studies suggest non‐random habitat selection (Pianka 1973; Smith and Ballinger 2001). Vegetation type (Muñoz et al. 2021), extent of native vegetation (Mulhall et al. 2022), prey availability in different vegetation types (McIntyre 2003), vegetation cover and height (Nemes et al. 2006), and vegetation composition (House and Spellerberg 1983; Tadevosyan 2007) can influence the habitat selection of lizard species on different spatial and temporal scales (Michael et al. 2017). Vegetation diversity can also increase the occupancy of snake species in grasslands (Stephenson 2022) and in agricultural habitats (Stephenson et al. 2024). Vertical structure, compositional diversity, and tussock height can also influence the habitat choice of grassland‐specialist reptiles (Mizsei et al. 2020).

The endangered Hungarian meadow viper ( *Vipera ursinii rakosiensis* ) inhabits fragmented grasslands in Hungary and Romania (Mizsei et al. 2018). Populations of these vipers historically exhibited high densities (Méhely 1912); nevertheless, current population sizes remain low, and the demographic trends are stagnant or decreasing despite substantial conservation efforts (Dankovics et al. 2004; Péchy et al. 2015). The remaining habitats of the viper also serve as refugia for numerous other ecologically valuable plant and animal species, designating the viper as an umbrella species whose conservation facilitates the persistence of diverse and characteristic grassland communities within the region (Roberge and Angelstam 2004; Vadász 2016; Králl 2020). In the light of evidence‐based conservation, monitoring and research efforts should focus on understanding the effects of different environmental and habitat features that influence the occupancy of this rare taxon (Sutherland et al. 2004). Vegetation cover and tussock height at the fine scale (Mizsei et al. 2020) and grassland management type (Mizsei, Budai, Moré, et al. 2023) are among the factors that are likely to affect the density of the viper, although there are certainly more non‐negligible underlying mechanisms to investigate. Vertical vegetation structural characteristics (such as the height of closed vegetation, maximum height of vegetation and leaf area) were measured in some parts of the study area using a standardised digital photography and image processing method (Mizsei et al. 2024, Oláh et al. 2025). Viper occurrence was higher in dense vegetation including higher plant elements and relatively open patches highlighting the complex influence of vertical phytomass structure on the habitat use of this subspecies (Mizsei et al. 2024). However, our understanding of how horizontal vegetation structure—reflected in plant community composition and species‐specific plant traits—relates to viper occurrence remains limited.

In this study, we explore how variations in vegetation composition, and species‐specific plant characteristics affect a grassland specialist reptile, the Hungarian meadow viper. This study aims to provide insights that inform conservation management practices tailored to preserve and enhance the habitats of this umbrella species. Our previous experiences suggest that vipers are more likely to be detected in certain types of grassland vegetation communities. Therefore, we test the hypothesis that plant composition affects both occupancy and density. We predict that habitat patches with higher plant species diversity (e.g., species richness) and functional diversity (e.g., height diversity) will exhibit higher density and occupancy values, while increased coverage of woody and alien species is expected to negatively affect these values.

## 2 Materials and Methods

### 2.1 Study Area and Sampling Design

**FIGURE 1:**
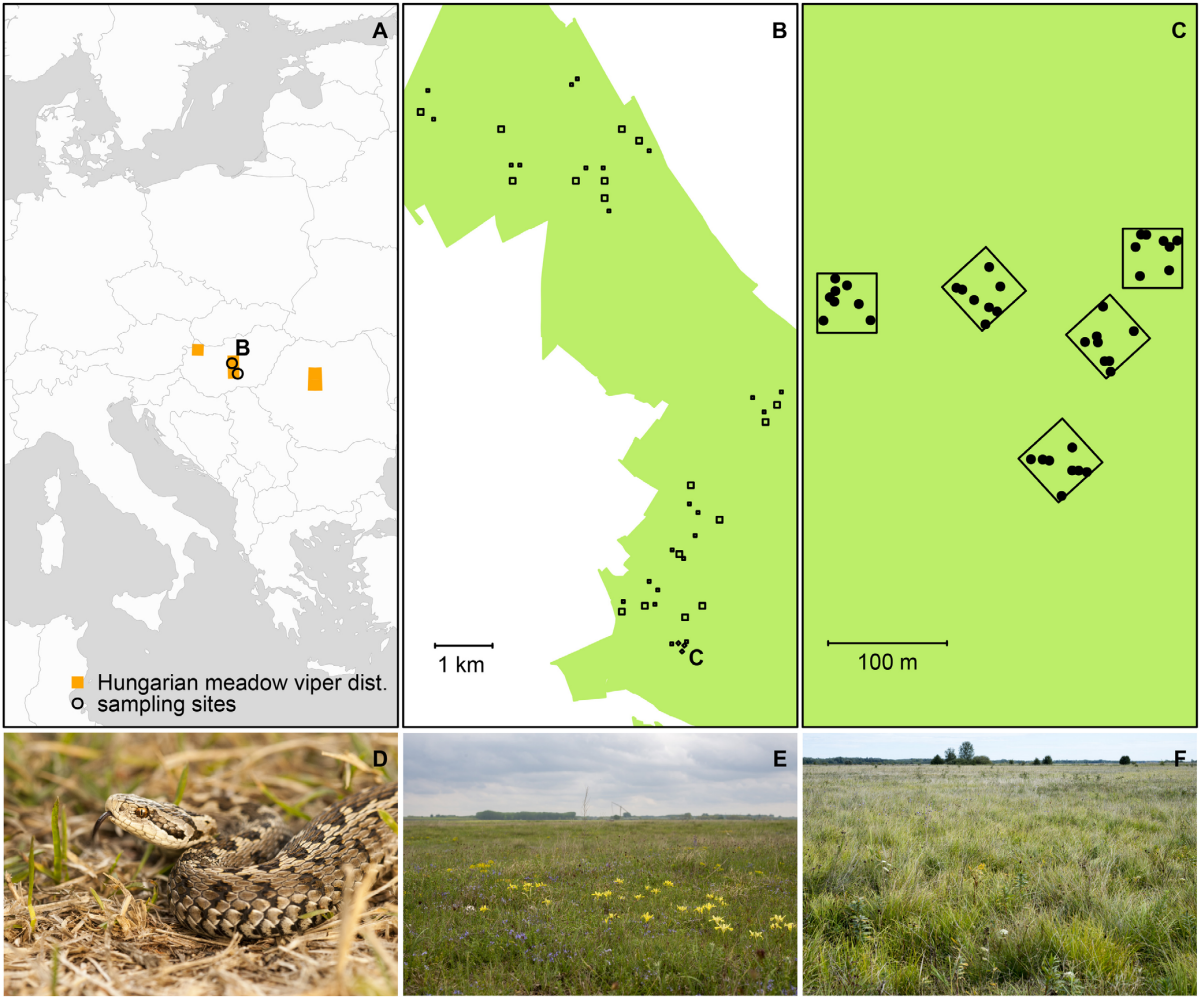
(A) Distribution of the Hungarian meadow viper (from Mizsei et al. 2018); (B) Examples for the locations of sampling plots for vipers at the HUKN2003 Natura2000 SCI; (C): Examples for the plant community sampling plots (1 × 1 m) within viper plots (50 × 50 m and 100 × 100 m); (D): A representative individual of a Hungarian meadow viper; panels E‐F: Examples of the sampled grassland habitats (photos by EM).

The study was conducted in two high conservation value grassland areas within Natura 2000 sites (HUKN20003 and HUKN20024), which are part of the European Union's network of protected areas, located in central Hungary, Europe (Figure 1). These grasslands exhibit a mosaic of several vegetation communities belonging to the priority habitat type Pannonian sand steppes (6260, EU Habitats Directive). These habitat mosaics encompass both primary and secondary grasslands, reflecting variations in land‐use history, including spontaneously regenerated or restored arable fields. A total of *n* = 59 sampling plots were established across the pastures (*n* = 44 on HUKN20003 and *n* = 15 on HUKN20024) for reptile data collection with two different plot sizes (50 × 50 m *n* = 36, 100 × 100 m *n* = 23). Two plot sizes were used to ensure reliable density estimations and optimal survey effort. Larger plots increased the probability of detecting individuals, but using only those would have reduced the number of sampling sites due to time and resource constraints. Sampling plots were semi‐randomly placed to encompass the range of grassland habitats of different aridity and naturalness (Figure 1). Within the sampling plots, plant composition was recorded in *n* = 8, 1 × 1 m subplots between 1 and 10 May 2024 (Figure 1). The weather conditions in the spring of 2024 resulted in unusually early vegetation development, with the spring–summer aspect occurring approximately 2 weeks earlier than usual. For this reason, the timing of the recordings can be considered ideal. Due to resource constraints, only the more species‐rich spring and early summer aspect was sampled, while the significantly different autumn vegetation aspect was not included.

### 2.2 Viper Surveys

Viper observations were collected during the spring of 2024 from the end of March to mid‐May, a period considered one of the seasons in which viper activity peaks due to post‐hibernation feeding and the mating season. We surveyed each sampling plot in *n* = 15 replicates. During each survey replicate, surveyors covered the whole area of the plot by random walking in east–west or north–south oriented straight lines located 5–10 m from each other. During surveys, the surveyor walked slowly (~2 km/h), stopping at least every 5 paces and looking around for reptiles. Survey duration varied from 5 to 30 min for 50 m × 50 m (0.25 ha) plots and 15–60 min for 100 m × 100 m (1 ha) plots, depending on the number of reptiles recorded and habitat complexity, with longer surveys conducted in denser vegetation. At least 30 min, but typically several hours or days, elapsed between repeated surveys at the same sampling quadrats. Surveys were conducted by experienced researchers or volunteers trained in viper detection. Previous analyses indicate that surveyor identity does not significantly affect viper detection probability. In addition to visual surveys, the sampling plots were surveyed at least once by a conservation detection dog unit trained for Hungarian meadow viper detection. We recorded the GPS position, time and individual data (i.e., sex, age, activity, and scalation pattern for further identification, not analysed here) of the detected reptiles for further research in the OpenBioMaps mobile application (Bán et al. 2022), including the metadata of the survey (start and end time, tracklog, surveyor id).

### 2.3 Observation Covariate

As temperature fundamentally influences the activity of reptiles (Falaschi 2021), we measured operative temperature during the surveys to model detection probability. Operative temperature is the environmental temperature that is available to an individual over time during thermoregulation (Shine and Kearney 2001). It cannot be calculated from commonly measured meteorological data due to the influence of radiation, surface temperature, wind speed, humidity, animal shape, and heat absorption. Therefore, to complement the most available air temperature (Kearney and Porter 2019), we applied *n* = 8 thermometers at each site (BTP‐06 temperature sensors connected to a BEL‐06 ecologger unit, Boreas Ltd., Hungary) placed in copper tubes in half‐shade of grass. The loggers recorded the operative temperature values at 2‐min intervals.

### 2.4 Plant Composition Variables

**FIGURE 2:**
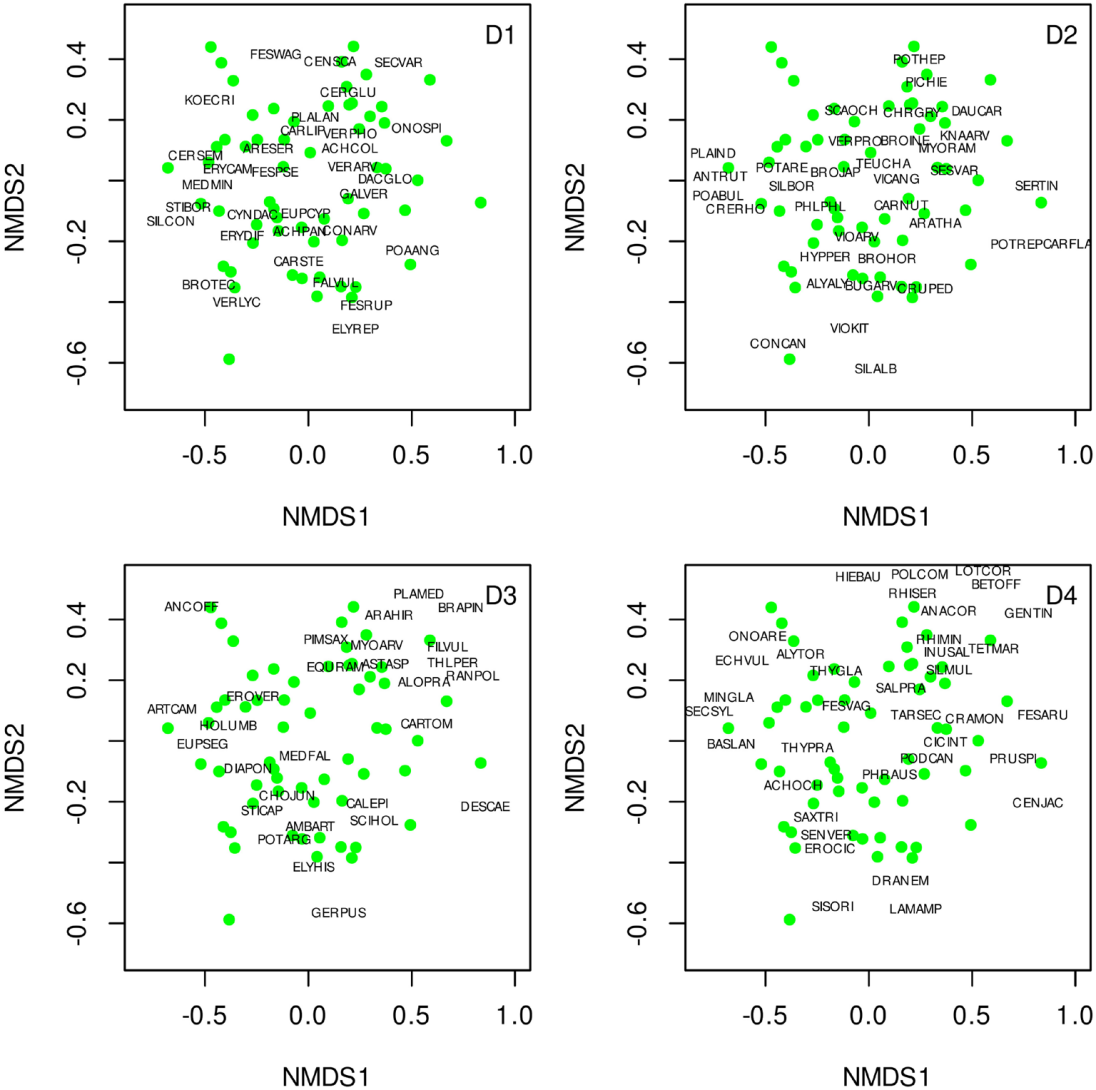
Non‐metric multidimensional scaling (NMDS) ordination of sampling plots based on cover data of plant species and Bray–Curtis dissimilarity. Each point represents the centroid NMDS position of the subplots within a respective site. Species arrows were passively projected onto the ordination diagram to indicate compositional gradients and the association between species. Species vectors are displayed in four panels (D1–D4), grouped by decreasing frequency across sites: D1 shows the most frequent 10% of species, D2 the next 10%, and so on, down to D4 (60%–70% frequency quantile). Labels show abbreviated species names based on the first three letters of the genus and species (e.g., *Festuca wagneri* = FESWAG).

Plant species composition was recorded as percentage covers of vascular plant species, with 0.1% as the lowest value. Skills of species identification and cover estimation were standardised across surveyors before sampling. We analysed the compositional gradients based on presence–absence data collected from the 1 × 1 m vegetation survey subplots. Non‐metric multidimensional scaling ordination was performed with the Bray‐Curtis index as a dissimilarity measure calculated based on species covers (Figure 2). We used the metaMDS function of the vegan (Oksanen et al. 2022) package for ordination analysis. The first two NMDS axes were used as predictors summarising plant community composition. NMDS reduced complex species data into ecologically interpretable gradients, reflecting key factors such as moisture and disturbance that influence both plant communities and viper habitat suitability. Although direct environmental measurements (e.g., soil moisture) were not consistently available across sites, NMDS captured their integrated effects as expressed through the vegetation. In addition, for each species, we provided the following properties based on Sonkoly et al. (2023) and Csiky et al. (2023): life cycle (annual or perennial), maximum plant height, functional group (graminoid [members of the Poaceae, Cyperaceae, Juncaceae, Iridaceae families], forb [non‐woody, dicotyledonous plants], shrub [woody plants]), residence status (native, alien). Using the data mentioned above, we calculated vegetation indices for each subplot (see Table 1). To assess multicollinearity, pairwise correlations among all explanatory variables were examined prior to model fitting. No strong correlations were detected; therefore, all variables were retained for model selection. The influence of residual collinearity was further reduced through information theoretic model selection.

**TABLE 1:** Summary statistics and description of vegetation indices used as site covariates in meadow viper occupancy and density modelling, calculated for sampling plots using the mean values of vegetation variables acquired from the subplots.

Variable	Mean + SD (min–max)	Description
Species richness	40.8 + 13.96 (11–68)	Total number of species detected
Annual‐perennial diversity ratio	0.63 + 0.19 (0.11–1)	H (annual)/[H (annual) + H (perennial)]
Height diversity	0.49 + 0.12 (0–1)	FD of plant height traits, standardised to 0–1
Height of plants	−0.1 + 0.09 (−0.22–0.61)	Mean of scaled (z‐transformed) max height trait
Graminoid‐forb ratio	0.38 + 0.23 (0–1)	Graminoid cover/(graminoid+forb cover)
Annual‐perennial ratio	0.93 + 0.13 (0.16–1)	Annual cover/(annual+perennial cover)
Moisture gradient (NMDS1)	0 + 0.4 (−0.78–1.06)	Ecological interpretations of NMDS positions of plots as centroids of subplot coordinates
Disturbance gradient (NMDS2)	0 + 0.29 (−0.67–0.66)	
Alien cover	0.01 + 0.05 (0–0.9)	Total cover of alien plants based on the list in CIT
Shrub cover	0 + 0.04 (0–0.65)	Total cover of common shrubs: *Crataegus monogyna* , *Prunus spinosa* , *Pyrus pyraster*

### 2.5 Modelling

We employed hierarchical models to analyse spatially and temporally replicated data on species with low and heterogeneous detection probabilities to assess the influence of vegetation variables on the occupancy and density of the studied viper populations. We applied the single‐season (single‐species) occupancy model of MacKenzie et al. (2002) and the N‐mixture model of Royle (2004) for density, as both are well‐suited for scenarios where data are collected over a short time frame without major environmental changes, making it appropriate for our study design. These models are using temporally replicated data to address imperfect detection, thereby reducing biases associated with false absences and non‐detections (MacKenzie and Royle 2005). Regarding N‐mixture model assumptions, our surveys were conducted within a single, relatively short sampling period (~50 days), during which demographic closure can reasonably be assumed. Based on telemetry data (Újvári and Korsós 1997), Hungarian meadow vipers exhibit limited movements (displacements less than 10 m), which are small relative to the size of our sampling plots (50 × 50 m and 100 × 100 m); consequently, occasional movements between nearby plots are unlikely to substantially bias density estimates.

Models were constructed within the Stan‐based modelling environment of the ubms package (Kéry and Royle 2020; Kellner et al. 2022) using the stan_occu and stan_pcount model fitting functions. In both model types, we used the same formula and parameters, except for the dependent variable, i.e., the viper observation data: in the occupancy models, we truncated the observation to 0–1 (0 = no detection or 1 = at least one detection during a survey replicate) and for the density models, we calculated the sum of observations during a survey replicate (ranging between 1 and 3).

We performed model selection on the combinations of the vegetation indices as site covariates and the mean operative temperature of a given survey as observation covariate. We generated the model selection formulas (*n* = 385) by including max. 4 variables in a model to avoid overparameterization. To test for non‐linear effects, we ran the models using the second‐degree polynomials of the site covariates. We included null models with and without observation covariates and site covariates as well. In each model, we included the natural logarithm of survey plot area (0.25 or 1 ha) as an offset of occupancy or density. The individual models were run with four replicates of Markov Chain Monte Carlo (MCMC) chains of 100,000 iterations. Model convergence was considered satisfactory if the Brooks‐Gelman‐Rubin statistic (R̂) was less than 1.1. We assessed the goodness of fit of the models using the gof function of the ubms package and considered a good fit with significance above 0.01.

Model selection was conducted using WAIC (Wattanabe‐Akaike information criterion, or the ‘widely applicable information criterion’) to identify the most parsimonious models. Models with ΔWAIC < 2 were considered to have substantial empirical evidence (Anderson and Burnham 2002). For the most robust models, we report the mean, standard deviation (SD), and 95% Bayesian credible interval (BCI) for each parameter. We considered a parameter significant when 90% of its proportion of the posterior distribution (*f*) excluded 0.

All data processing and analysis were conducted in the R statistical environment (R 4.1.3, R Core Team 2022).

## 3 Results

### 3.1 Plant Communities

A total of 314 plant taxa were recorded during the 480 plant community samples. The average number of species in the 1 m^2^ subplots was 18.9 (minimum 3, maximum 40).

The NMDS ordination was optimised at 0.2503 stress value. The 1st axis corresponded with a moisture gradient, reflecting a transition from dry to wet grassland types. Low NMDS1 values were associated with very dry and dry grassland species (e.g., *Stipa borysthenica, Koeleria cristata*), mid‐range values with mesic grassland species (e.g., *Achillea pannonica* , *Medicago falcata* ), and high values with wet meadow species (e.g., *Serratula tinctoria* , *Carex flacca* ) (Figure 2). NMDS1 scores showed a strong correspondence with Á‐NÉR habitat types (General National Habitat Classification System; Bölöni et al. 2007), which represent established classifications of Hungarian grassland vegetation. Open sand steppes (dry) were positioned at the lower end of the gradient, closed sand steppes (mesic) clustered near the center, and Molinia meadows together with other wet grassland types occurred at the upper end. Axis 2 reflected a disturbance or vegetation openness gradient with less disturbed and less open subplots towards more positive values and more disturbed, more open subplots at the more negative values.

### 3.2 Viper Observations

In *n* = 59 sampling plots, we conducted *n* = 15 survey replicates resulting in a total of *n* = 885 surveys. We detected the meadow viper in 3.8% of the surveys (*n* = 37 observations). The species was detected at least once in 28.8% of the sampling plots (*n* = 17 sites, naїve occupancy, ψ^ = 0.29).

### 3.3 Influence of Plant Variables on Viper Occupancy and Density

According to the model selection, the best model included the moisture related gradient (NMDS1 axis), species richness, graminoid‐forb ratio, and height of plants as the primary variables influencing viper occupancy as well as density (Table 2). Moisture gradient and species richness were present in all of the 10 most parsimonious models based on the lowest ΔWAIC values. The best models with ΔWAIC < 2 also included shrub cover, height diversity, and annual‐perennial diversity ratio as predictor variables (Table 2). Moisture gradient, species richness, and graminoid‐forb ratio were considered the most important variables explaining occupancy. In the case of density, apart from these three variables, height of plants was also among the strongly supported factors mainly determining the density of the meadow viper (Table 3).

**TABLE 2:** Model selection results for site covariates based on the Watanabe–Akaike Information Criterion (WAIC). The table presents only those models with ΔWAIC < 2, which were considered to have substantial empirical support and represent the most robust occupancy and density models. WAIC values in parentheses correspond to the best‐fitting model (i.e., with the lowest WAIC).

Site variables							ΔWAIC	
							Occupancy	Density
Moisture	+	Species richness	+	Graminoid‐forb ratio	+	Height of plants	0 (261.18)	0 (231.09)
Moisture	+	Species richness	+	Height of plants			0.35	1.75
Moisture	+	Species richness	+	Height of plants	+	Shrub cover	1.18	
Moisture	+	Species richness	+	Graminoid‐forb ratio	+	Shrub cover	1.36	
Moisture	+	Species richness	+	Height diversity			1.58	
Moisture	+	Species richness	+	Graminoid‐forb ratio			1.65	
Moisture	+	Species richness	+	Height of plants	+	Height diversity	1.77	
Moisture	+	Species richness	+	Graminoid‐forb ratio	+	Height diversity	1.79	
Moisture	+	Species richness	+	Graminoid‐forb ratio	+	Annual‐perennial diversity ratio	1.98	

**TABLE 3:** Summary of site covariates included in the best‐supported occupancy and density models. Variable importance was assessed based on the frequency of inclusion of each covariate across top‐performing models (ΔWAIC < 2), expressed as a percentage. Parameter estimates represent the posterior median values with associated 95% Bayesian credible intervals (BCI; defined as the 2.5th and 97.5th percentiles of the posterior distribution) and the proportion of the posterior distribution (*f*) supporting a positive or negative effect is also reported. Covariate effects were considered strongly supported when their 95% BCI did not overlap zero or when the posterior proportion exceeded 90%; these are indicated in bold.

Model	Site variable	Variable importance	Estimate (BCI)	*f* value	*R* hat
Occupancy	**Moisture gradient (NMDS1)**	**1.00**	**1.288 (−0.033, 2.839)**	**0.97**	**1.00003**
	**Species richness**	**1.00**	**−1.563 (−3.054, −0.356)**	**0.99**	**1.00005**
	**Graminoid‐forb ratio**	**0.56**	**−0.89 (−2.058, −0.011)**	**0.98**	**1.00000**
	Height of plants	0.44	−0.866 (−2.487, 0.555)	0.89	1.00003
	Height diversity	0.33	−0.808 (−2.376, 0.519)	0.89	1.00001
	Shrub cover	0.22	−0.851 (−3.683, 0.97)	0.84	1.00001
	Annual‐perennial diversity ratio	0.11	−0.586 (−1.926, 0.654)	0.84	1.00000
Density	**Moisture gradient (NMDS1)**	**1.00**	**1.87 (0.956, 2.863)**	**1.00**	**1.00007**
	**Species richness**	**1.00**	**−1.021 (−1.834, −0.245)**	**1.00**	**1.00005**
	**Graminoid‐forb ratio**	**0.50**	**−0.592 (−1.298, −0.033)**	**0.98**	**1.00002**
	**Height of plants**	**1.00**	**−1.481 (−2.887, −0.265)**	**0.99**	**1.00001**

**FIGURE 3:**
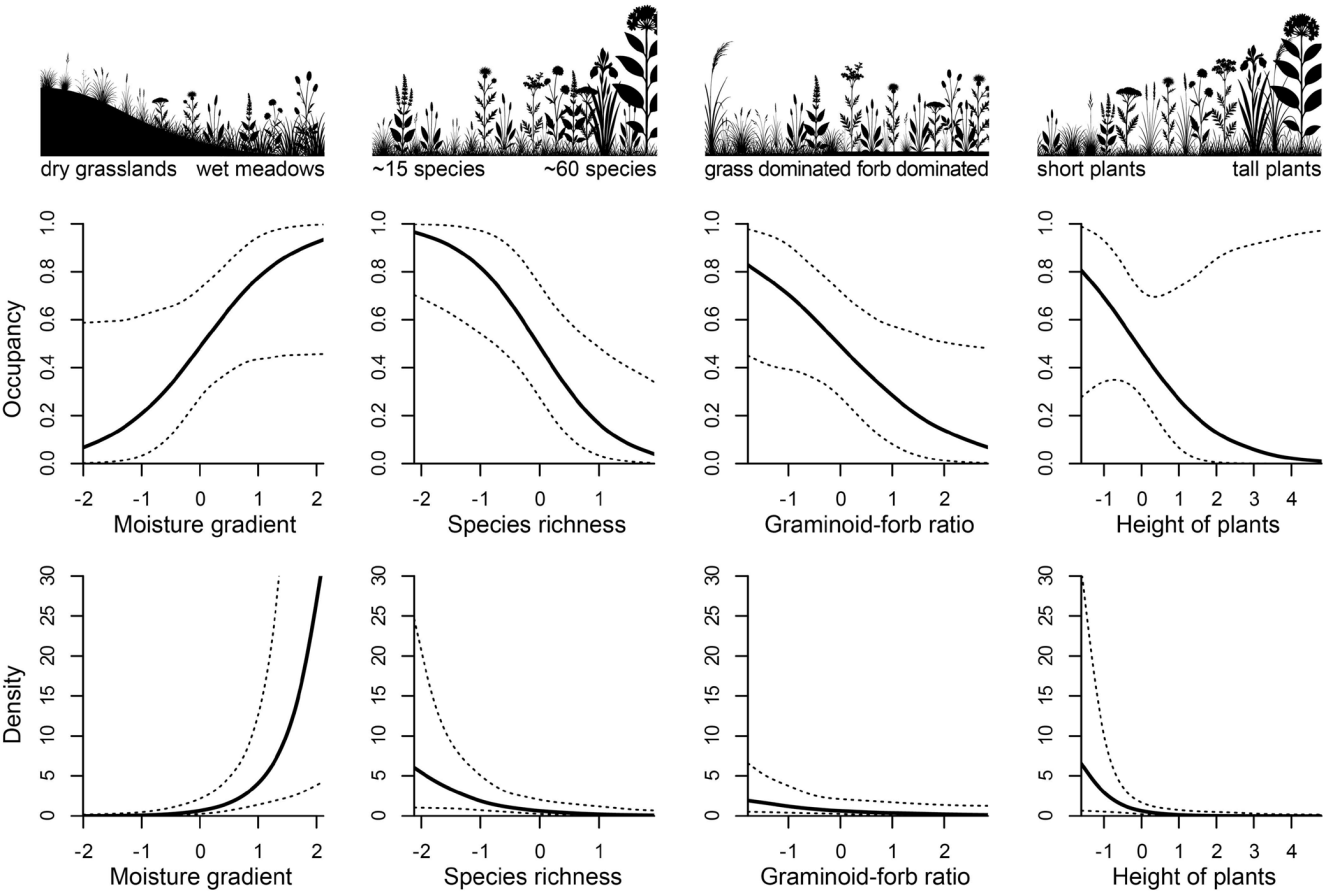
Marginal effect of vegetation variables on Hungarian meadow viper ( *Vipera ursinii rakosiensis* ) occupancy and density. Dashed lines indicate 95% confidence intervals.

The models with a ΔWAIC < 2 showed that the NMDS1 axis, representing a moisture gradient, showed a rather positive effect on both the occupancy and density of the Hungarian meadow viper; thus, vipers used wetter meadows more frequently than drier meadow patches. Species richness, graminoid‐forb cover, and height of plants all had a negative effect on occupancy and a slight negative effect on density (Figure 3). Hungarian meadow viper occupancy and density were higher in meadows with less and typically lower growing plant species, including more graminoids against less forbs.

### 3.4 Estimated Viper Occupancy and Density

**FIGURE 4:**
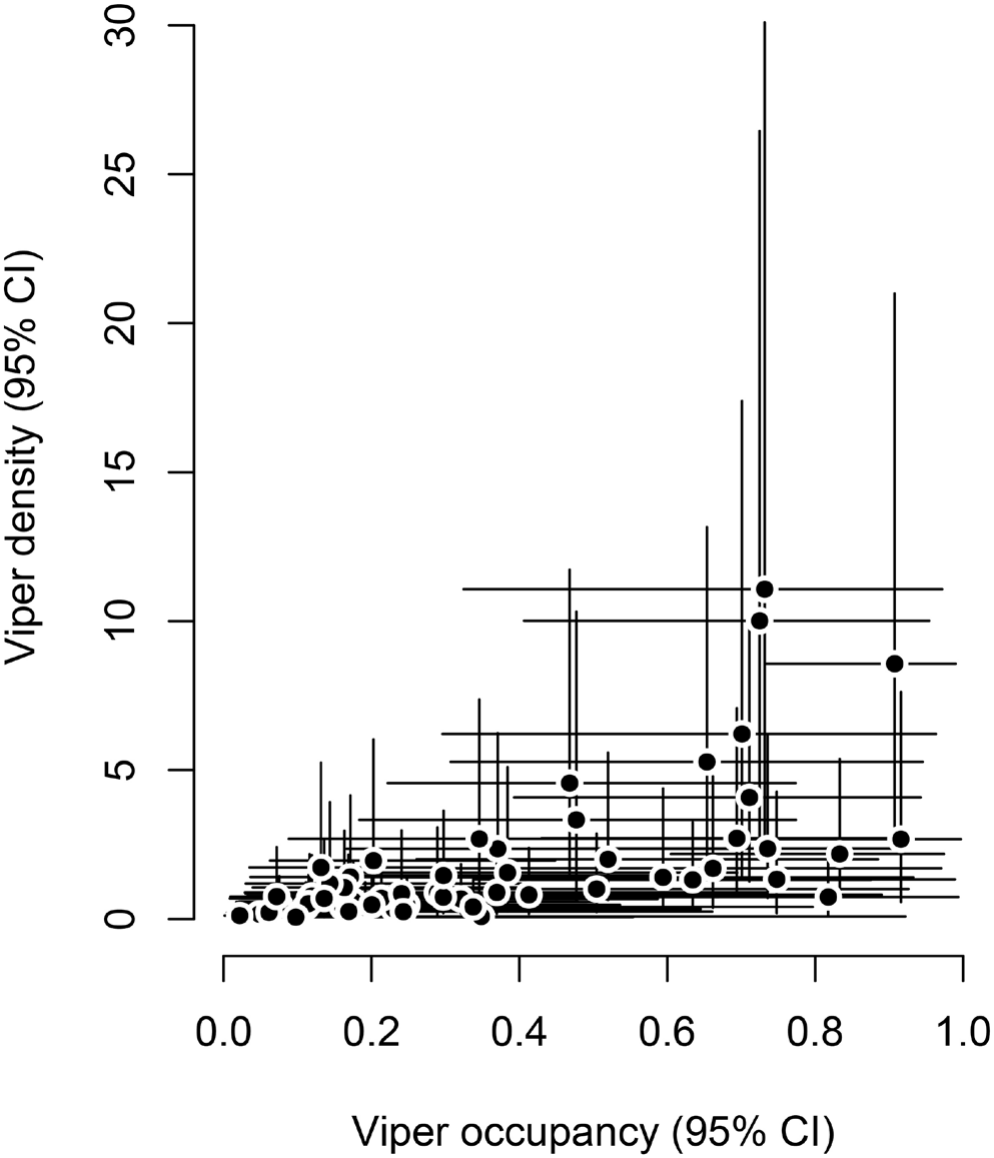
Relationship between predicted site‐level occupancy and density (individual/ha) of the Hungarian meadow viper ( *Vipera ursinii rakosiensis* ). Estimates are posterior means derived from hierarchical occupancy and N‐mixture models, with 95% credible intervals shown as vertical (density) and horizontal (occupancy) lines.

According to our models, the estimated occupancy of the Hungarian meadow viper was 0.36 ± 0.011 (±SE, CI = 0.04–0.9), while the mean density was 1.75 ± 0.21 individual/ha (±SE, CI = 0.11–9.43). Density and occupancy estimates were correlated (Spearman's *r* = 0.7, *p* < 0.0001), meaning that our estimates and effects were consistent across our two response variables (Figure 4).

## 4 Discussion

Our results showed that plant species composition influences the occupancy and the density of the Hungarian meadow viper in a similar way. Vipers are more likely to occur in more humid meadows compared to drier grasslands, as moisture related gradient indicated by NMDS1 proved to be one of the most important variables in their habitat use. Higher ratio of graminoids to forbs and lower plant species richness positively influences occupancy and density of the vipers, while they also seem to occupy meadows dominated by characteristically lower‐growing plant species. The factors determining density and occupancy did not differ significantly. Overall, these findings underscore the complex habitat requirements of this endangered reptile taxon and highlight the importance of specific plant community characteristics for its persistence.

Several studies have examined the effects of different habitat characteristics on the occurrence of reptiles; although, most have not considered fine‐scale plant species composition and plant functional traits. Hungarian meadow viper abundance was negatively affected by the compositional diversity of vegetation in a previous study; however, data were obtained from a smaller spatial scale, and different methods were used to measure vegetation variables (Mizsei et al. 2020). In earlier studies, plant diversity had no impact on grassland snake communities (Glass and Eichholz 2022), while native plant species richness had a positive effect on the abundance of a skink species in an Australian temperate woodland (Brown et al. 2011). In contrast to these, species richness proved to negatively influence the occupancy and density of the Hungarian meadow viper considering not only native but all present plant species. Occupancy and density are often related in other ecological studies as they mainly depend on sampling scales (Holt et al. 2002; Steenweg et al. 2018). Glass and Eichholz (2022) also measured forb and grass cover, similarly to our variable, graminoid‐forb ratio, but showed opposite results in the case of grassland snake communities as they showed higher abundance and diversity when a higher ratio of forbs was measured against grasses. Generally, vegetation height and cover values are used instead of plant traits (i.e., the maximum plant height of species in our case) in relation to reptile abundance and diversity (House and Spellerberg 1983; Garden et al. 2007; Bateman and Merritt 2020), despite the determining role of plant traits and vegetation structure in ecosystem functioning (Díaz and Cabido 2001). With the increasing number of accessible databases on plant functional traits, a growing number of studies can be expected on the impact of such traits on animal occupancy and density in the near future. Shrub cover had a negative effect on occupancy and density in our study, but it was not included in the best models. Canopy cover and shrubs mostly have a positive effect on reptile abundance, as they provide shelter and feeding sites for the individuals, especially in wooded grasslands (Brown et al. 2011; Michael et al. 2015; Owen et al. 2024). The species examined in these studies, however, seem to require different microhabitats than the grassland‐specialist Hungarian meadow viper, as their abundance was higher in the presence of woody plants and shrubs, while our studied grasslands have generally low shrub coverage. The effects of increasing aridity and more frequent, prolonged droughts on biodiversity are in the focus of numerous studies (Frank et al. 2014; Peterson et al. 2021; Martín et al. 2023). These studies were conducted in drier habitat types, but our study demonstrated the importance of humidity in drying grasslands as well, as supported by the positive effect of the NMDS1 axis.

The moisture gradient had the most significant impact on the occupancy and density of the Hungarian meadow viper, which indicates that the occupancy and density of the vipers were higher in grassland patches with a more wet‐adapted plant species composition compared to drier habitat patches. NMDS1 revealed a moisture‐driven gradient in the studied grasslands, showing a transition from dry sand steppe species through mesic species to wet meadow species, closely reflecting established Hungarian Á‐NÉR habitat classifications. The sampling area, which covers the core habitats where the majority of the Hungarian meadow viper populations are found, consists of heterogeneous grasslands with varying microtopography and hydrological conditions, a significant part of which was much wetter in the past and was often periodically covered with water. The draining of the landscape in the last century and the irregular and drastically reduced rainfall experienced in recent years due to climate change have severely affected the ecosystem of the habitat of the vipers (Kovács 2008; Jánosi et al. 2023). Currently, severe droughts seem to be the most significant threat to these grasslands, and possibly also to meadow vipers, as vipers tend to withdraw to the wettest patches of the landscape (Ladányi et al. 2015). Reduced water availability drives a shift in plant species composition, abundance, and functional properties (Tölgyesi et al. 2016; Ónodi et al. 2025). Through changes in vegetation structure, the availability of prey, hiding places, and basking spots for vipers can be reduced. In addition, the habitat selection of vipers can be directly influenced by humidity, as research on evaporative water loss has shown (Guillon et al. 2014). NMDS2 captured only a weak degradation gradient and showed no direct association with Hungarian meadow viper occupancy or density, although other variables may still link habitat disturbance and degradation to viper habitat use. Species richness was negatively associated with both occupancy and density, which may initially appear counterintuitive, as higher species richness generally implies greater habitat complexity, an attribute typically considered important for the habitat selection of the Hungarian meadow viper. However, the studied grasslands are mostly meadows in a highly natural state with a mean of 18.9 plant species/m^2^. This can be considered a high species richness at fine spatial grain since the habitat‐specific species pool is limited. Even higher fine‐scale richness in the studied system can indicate excessive disturbance, promoting the establishment of ruderal species, negatively affecting the occupancy and density of the viper (Pruchniewicz 2017). A similar factor may also be behind the negative impact of the plant height variable, as disturbance‐tolerant species, including invasive and alien species, are usually taller and functionally different from the dominant species of more natural patches (Ordonez et al. 2010). The high value of this variable does not necessarily reflect a tall vertical vegetation structure, but rather the occurrence of species with tall reproductive shoots or other structures that are not uniformly detectable across all species at the time of the survey. Our previous research showed that the maximum vegetation height itself positively affects viper occurrence (Mizsei et al. 2024). A species‐rich area with more plant species might indicate a nutrient‐rich, overgrazed habitat patch, where while characteristic species of these grasslands are still present, disturbance‐tolerant weeds also appear. Another potential explanation for the negative relationship between species richness and viper occurrence is that the mesic and species‐rich sandy meadow steppe grasslands (H5b habitat type, typical of the study area) may no longer provide sufficiently moist conditions. Consequently, vipers are more likely to be detected in slightly less species‐rich but wetter patches. The most probable reason behind the importance of the forb‐graminoid ratio in the occupancy and density of the meadow vipers is their sensitivity to habitat conversion and habitat quality. The Hungarian meadow viper populations managed to survive in habitats that have not been subject to significant conversion and disturbance in the recent past (Újvári et al. 2002). In general, the successional process of grasslands results in an initial dominance of forb species that are increasingly replaced by grasses over time (Ladouceur et al. 2023). The preference of the vipers towards the higher ratio of graminoid cover might show that those habitats were not recently affected by ploughing, overgrazing, or any other harmful disturbance (Søndergaard et al. 2025). Moreover, vegetation structure elements preferred by vipers, for instance tussocks, are mainly shaped by grass species (Peach and Zedler 2006; Mizsei et al. 2020).

Despite the significant sampling effort, certain limiting factors need to be understood when interpreting the results. First, plant composition surveys were limited to a single spring season. Consequently, the restricted spatial and temporal scope may limit the generality of the findings, as viper–habitat relationships may differ among seasons, years, or habitats. Second, during field sampling, we did not record vegetation structure per se, but plant species composition, and inferred vegetation structure from the known traits of species obtained from literature data or databases. These sources contain averages or maxima of the species during their entire life span, which often differ from what is realised in a particular community at a particular time, due to site‐specific physiological limitations, herbivory or species‐specific phenology. Although we did not directly measure habitat moisture and disturbance gradients, the NMDS axes seemed to capture these environmental patterns to a considerable extent. Resource constraints limited our ability to complete a more thorough survey of vegetation repeated several times during the year. However, plant communities were sampled during spring and early summer, which is the peak of viper activity due to the mating season, and also the main period for vegetation development, when most plant parts are visible above the ground. Therefore, we believe that our sample still represents the studied vegetation sufficiently, especially as it is experienced by vipers in their spring and early summer activity period. Given the extremely low detectability of the Hungarian meadow viper, 37 observations can provide a relatively representative insight into the habitat use of this taxon in the Kiskunság region, which is home to the majority of the populations of these snakes in Hungary. It is important to note that the fine‐scale, 1 × 1 m quadrats used for plant community sampling included shrubs and alien species only in very low numbers. Given their low probability and minimal cover values in the dataset, these factors are unlikely to consistently influence viper occurrence, which likely explains their exclusion from the most parsimonious models. Fortunately, this suggests that excessive shrub encroachment and the spread of alien species have not yet critically impacted all viper habitats due to the regular and appropriate management of these grasslands. However, at broader spatial scales, increasing shrub and alien plant cover represents a potential threat to the meadow viper populations. Future research should investigate whether the effects are different in the autumn season and moreover, it is worth comparing the results with grasslands where the Hungarian meadow viper has already become extinct. Comparing the plant species composition with the standardised vertical vegetation structure measurement of the same season would also give insights into the complex habitat requirements of the viper. While numerous studies have investigated the influence of various vegetation variables on animal species, inconsistencies in variable names, definitions, and measurement methods limit comparability. Therefore, reviewing and standardising these variables is essential for enhancing the comparability of results and supporting future research.

Our findings emphasise the role of plant species composition in the habitat selection of the Hungarian meadow viper; however, it is likely that the effect of plant species composition on vipers is mainly indirect. The plant species inhabiting a grassland patch determine the vertical structure of vegetation, which is important for thermoregulation and for finding shelters from predators, while it also influences prey availability, which also shapes the habitat preference of the taxa (Mizsei et al. 2024). We suggest that our most important result is that occupancy and density of the vipers were higher in wetter meadows as a potential response to the droughts of recent years. The formerly waterlogged grasslands of the Kiskunság region have undergone significant transformations in landscape structure, vegetation composition, and species assemblages due to drainage ditches and canals that were established in the last century, compounded by increasingly irregular, sparse, and reduced rainfall in recent years (Tölgyesi et al. 2016; Orbán et al. 2023; Erdős et al. 2024). Due to climate change, the situation is likely to continue to deteriorate in the future, and the valuable habitats and species of the region will have to be preserved in increasingly arid conditions, so urgent water management interventions are needed in the landscape in order to save these grasslands (Kovács et al. 2017; Báder 2023). The results also show that the vipers are possibly sensitive to habitat quality as they are more likely to inhabit grasslands that have not been affected by conversions or excessive disturbances in recent times. Overgrazing, agricultural cultivation, and mowing should be avoided, which allows shaping a grassland community mostly characterised by native grasses (Mizsei, Budai, Moré, et al. 2023; Szentes et al. 2024). In addition, total abandonment of habitats can also be harmful, as the appearance of shrubs and less demanding, invasive, and alien species are likely in some unmanaged grassland fragments (Lenda et al. 2012; Pruchniewicz 2017). In the case of this endangered taxon, rare and moderate cattle grazing proved to be the most effective habitat utilisation method for maintaining habitat quality and a mosaic structure of habitat types but should be particularly limited for drier sandy grasslands (Mizsei, Budai, Moré, et al. 2023; Rák et al., n.d.). To expand the populated area of vipers with new habitats, we recommend reconstructing abandoned ploughed fields by introducing propagules to target areas (Reis et al. 2023). This may accelerate the establishment of an optimal plant species composition for vipers and their prey animals, thereby facilitating their early colonisation of these grassland habitats (O'Reilly‐Nugent et al. 2024).

## 5 Conclusions

We demonstrated that, in addition to the vertical structure of vegetation, the horizontal composition of plant communities also influences the occupancy and density of the grassland‐specialist Hungarian meadow viper. Nonetheless, viper habitat use is inherently complex, driven by the interplay of multiple interacting factors. Our findings emphasise the vulnerability of this endangered subspecies, as vipers are likely sensitive to habitat drainage, land conversion, and excessive disturbance. Consequently, occupancy and density are greater in wetter meadows at later successional stages, characterised by native graminoid assemblages with less ruderal, disturbance‐tolerant species. However, given the limited spatial and temporal sampling, the effects should be interpreted cautiously. Conservation measures should therefore prioritise the protection of the remaining grasslands with complex plant composition, while avoiding excessive disturbances (e.g., ploughing, overgrazing). Furthermore, urgent efforts are required to enhance water availability and retention in the landscape, given the increasing impacts of climate change on these ecosystems. Finally, while our vegetation‐based approach may be applicable to other grassland‐specialist species, it necessitates considerable sampling effort, as well as the definition and standardisation of vegetation variables and survey methodologies to enable broader application in future research.

## Author Contributions

**Mátyás Budai:** data curation (equal), formal analysis (supporting), investigation (equal), writing – original draft (lead), writing – review and editing (lead). **Gergő Rák:** investigation (equal), writing – review and editing (equal). **Bálint Wenner:** investigation (equal), writing – review and editing (equal). **Attila Móré:** investigation (equal), writing – review and editing (equal). **Barnabás Bancsik:** investigation (equal), writing – review and editing (equal). **Bence Nagy:** investigation (equal), writing – review and editing (equal). **Gergő Kovács:** investigation (equal), writing – review and editing (equal). **Márton Szabolcs:** investigation (equal), writing – review and editing (equal). **Zsolt Ladnyik:** investigation (equal), writing – review and editing (equal). **Csaba Molnár:** investigation (equal), writing – review and editing (equal). **Zsófia Eszter Guller:** investigation (equal), writing – review and editing (equal). **Attila Lengyel:** conceptualization (equal), writing – review and editing (equal). **Csaba Vadász:** conceptualization (equal), writing – review and editing (equal). **Edvárd Mizsei:** conceptualization (equal), formal analysis (lead), investigation (equal), writing – review and editing (equal).

## Conflicts of Interest

The authors declare no conflicts of interest.

## Data Availability

All the required data are uploaded as Supporting Information.
